# Enhancing rehabilitation in stroke survivors: a deep learning approach to access upper extremity movement using accelerometry data

**DOI:** 10.3389/frai.2025.1547127

**Published:** 2025-11-05

**Authors:** Tan Tran, Lin-Ching Chang, Peter S. Lum

**Affiliations:** ^1^Department of Computer Science, The Catholic University of America, Washington, DC, United States; ^2^Department of Informatics, New Jersey Institute of Technology, Newark, NJ, United States; ^3^Department of Biomedical Engineering, The Catholic University of America, Washington, DC, United States

**Keywords:** deep learning, upper extremity, stroke, rehabilitation, functional use

## Abstract

Upper Extremity (UE) rehabilitation is crucial for stroke survivors, aiming to improve the use of the paretic UE in everyday activities. However, assessing the effectiveness of these treatments is challenging due to a lack of objective measurement tools. Traditional methods, such as clinician-rated motor ability or patient self-reports, often fail to measure UE performance in real-life settings accurately. Evidence suggests that currently used clinical assessments do not reliably capture actual UE use at home or in the community. This study investigates the application of Convolutional Neural Networks (CNNs) combined with Dense layers using accelerometry data from wrist-worn sensors to classify functional and non-functional UE movements of stroke survivors. Two types of models were developed: one trained on data from individual subjects (intrasubject model) and another trained on data across all subjects (intersubject model). The intrasubject model for the paretic UE achieved an average accuracy of 0.90 ± 0.05, while the intersubject model reached an accuracy of 0.79 ± 0.06. When incorporating signals from the non-paretic arm, the intersubject model’s accuracy improves to 0.88 ± 0.10. Notably, this method utilized raw accelerometry data, eliminating the need for manual feature extraction, which is commonly required in traditional machine learning, and yielded higher accuracy than previously reported methods. This proposed deep learning approach incorporates CNNs with Dense layers, offering a cost-effective and adaptable method for monitoring UE functionality in real-world settings. The results from this study have the potential to inform the development of personalized rehabilitation strategies for stroke survivors, offering valuable insights for clinical practice.

## 1 Introduction

A stroke occurs when blood flow to the brain is disrupted, either by a blockage that prevents oxygen and nutrients from reaching brain cells or by the rupture of a blood vessel. This interruption can rapidly damage or kill brain cells. Strokes are a significant global health issue, ranking as the third leading cause of death worldwide and contributing significantly to cardiovascular disease-related fatalities (Feigin et al., 2021). In the United States alone, approximately 795,000 people suffer from strokes each year, with about two-thirds surviving but often requiring extensive rehabilitation (Sun et al., 2012). A common consequence of stroke is impaired upper extremity (UE) function, typically due to hemiparesis, which manifests as reduced arm movement and altered muscle tone (Lang et al., 2013). Effective rehabilitation of UE functions is crucial for restoring movement and functionality, enabling stroke survivors to regain independence and improve their quality of life (Barreca et al., 2003).

The International Classification of Functioning, Disability and Health (ICF) defines domains affected by a stroke: body functions, activities, and participation (Bernhardt et al., 2017). Stroke can affect body functions, such as impairments in muscle strength or joint range of motion. The activities domain encompasses functional tasks that rely on the proper integration of multiple body functions, such as reaching to grasp or walking. Participation is the end goal of rehabilitation and includes return to work, social, or recreational activities. Spanning these domains are the concepts of capacity and performance (Kwakkel et al., 2023). Capacity is measured by the ability to perform tasks, usually in controlled situations, such as during a clinical test where the patient is asked to pick up an object with the paretic arm. Capacity assessments can be augmented with kinematic analysis of the tasks using optical motion capture (Alt Murphy et al., 2011). Data from wrist-worn Inertial Measurement Units (IMUs) during execution of a clinical capacity scale can predict scores from trained clinicians (Werner et al., 2022), and IMUs can potentially replace optimal motion capture systems in assessing movement quality (Unger et al., 2024). However, many factors can limit spontaneous real-world arm use (performance) even if capacity seems adequate, such as the effort required to use the paretic limb compared to compensation with the less-affected arm. Capacity measured in the clinic often does not correlate with real-world performance (Lundquist et al., 2022).

To address the limitations of traditional methods, emerging technologies have been developed to provide more objective and accurate measurements of UE function. One such advancement is the use of IMUs, which are small, wearable sensors that track movement through accelerometers and gyroscopes (Papi et al., 2015; Parkka et al., 2006; Uswatte et al., 2000; Uswatte et al., 2005; Rand and Eng, 2015; Sengupta et al., 2024). IMUs can be placed on various body parts to collect detailed movement data over extended periods, both in clinical settings and during daily activities (Bochniewicz et al., 2017; Unger et al., 2024).

Prior research has demonstrated the potential of accelerometry data combined with machine learning algorithms to classify and analyze UE movements. Studies have shown that IMUs can effectively capture the nuances of arm movements, providing valuable insights into motor function and the progress of rehabilitation. For instance, research has utilized accelerometry data to distinguish between functional and non-functional movements, providing a more objective assessment than traditional methods (Tran et al., 2018; Lum et al., 2020; Pohl et al., 2022).

In addition to their use in classification, accelerometry outputs can also be used to generate clinically meaningful assessments. The earliest metric proposed was activity counts, which are calculated by summing the periods of time during which the filtered acceleration magnitude exceeds a predefined threshold. However, in its original implementation, this metric has poor specificity for detecting functional use of the limb (Subash et al., 2022; Lum et al., 2020). Optimal count thresholds can be derived from labeled accelerometry data, achieving an accuracy of 80% in predicting functional limb use (Pohl et al., 2022). Once functional use is estimated for each limb, the relative use of the upper extremities can be visualized graphically, and metrics that capture the limb asymmetries can be calculated (David et al., 2021). If additional IMUs are used on the upper limb and trunk, they can be used to estimate clinical parameters of interest during execution of functional tasks, such as elbow angle, shoulder angle, and trunk movement (Unger et al., 2024).

Machine learning has also been widely used in upper extremity (UE) assessment (Geed et al., 2023; Tozlu et al., 2020) to provide objective, scalable, and quantifiable measures of motor function, offering more accurate evaluations compared to traditional methods. Techniques such as Random Forest (RF), Support Vector Machines, Logistic Regression Classifiers, and traditional neural networks have been employed to classify functional and non-functional movements from sensor data (Ghannam and Techtmann, 2021; Arikumar et al., 2022), often acquired with IMUs or accelerometers, to monitor stroke recovery progress. These models have the potential to automate the assessment process, reduce reliance on subjective clinical evaluations, and enable continuous monitoring in real-world environments. However, earlier approaches often required labor-intensive manual feature engineering, such as selecting specific attributes like velocity or joint angles, limiting these models’ flexibility, adaptability, and generalizability. Furthermore, hand-selected features may fail to fully represent the complexity of movement patterns, resulting in less accurate assessments. Consequently, models based on manually extracted features often struggle to generalize effectively across diverse patient populations and movement contexts. According to (Lum et al., 2020), a machine learning model was used to report an accuracy of 74.2% from the intersubject model with 10 stroke survivors.

The validity of these classification approaches has also been demonstrated through comparisons with established clinical measures. The concurrent validity of the functional/non-functional ratio against several clinical outcomes was previously published (Geed et al., 2023). The ratio was found to be highly correlated with the Action Research Arm Test (ARAT), Fugl-Meyer, 9-hole Peg Test, and the Motor Activity Log. Another study by (Pohl et al., 2022) demonstrated the validity of distinguishing functional from non-functional movements using IMU data. By comparing conventional thresholding, optimal thresholds, and a logistic regression classifier, the authors found that both the optimal thresholding and logistic regression classifier methods achieved approximately 80% accuracy in inter-subject model and outperformed conventional thresholding.

Convolutional Neural Networks (CNNs) have become increasingly prominent in medical and healthcare applications due to their ability to process and analyze complex data. In healthcare, CNNs have been used for tasks such as medical image analysis, disease detection, and patient monitoring (Shen et al., 2017; Ravi et al., 2017; Hossain et al., 2023). The application of CNNs to movement assessment, especially in stroke rehabilitation, is an emerging field with significant potential (Szczesna et al., 2020).

In this study, we investigated the application of a deep learning architecture that combines CNN and dense layers in stroke rehabilitation. CNN layers, which consist of convolutional and pooling operations, are designed to extract spatial or local patterns from input data, such as images or sensor signals. Dense layers, also known as fully connected (FC) layers, receive the extracted features from CNNs and learn complex, non-linear combinations to perform the final prediction. In a Dense layer, every neuron is connected to all neurons in the preceding layer through learnable weights (Ram and Reyes-Aldasoro, 2020). In the context of upper extremity movement classification, Dense layers serve to map the temporal–spatial features extracted by preceding convolutional layers to output classes representing functional and non-functional arm movements. The use of CNNs offers significant advantages for movement assessment. These models can automatically extract salient features directly from raw input data, minimizing the need for manual feature engineering. This capability is especially beneficial when processing large and complex datasets, such as those collected by inertial measurement units (IMUs) (Ordonez and Roggen, 2016). By learning hierarchical patterns and subtle correlations within the data, CNNs can produce more accurate and reliable movement classifications than traditional analysis methods (LeCun et al., 2015).

However, the use of CNNs in movement assessment also presents challenges. One major challenge is the requirement for large datasets to train the models effectively, which can be difficult to obtain in clinical settings. Additionally, the complexity of CNNs can lead to overfitting, where the model performs well on training data but poorly on unseen data. Regularization techniques, such as dropout and batch normalization, are often employed to mitigate this issue. Despite these challenges, the integration of CNNs with IMUs data holds promise for advancing UE assessment and improving rehabilitation outcomes for stroke survivors.

This study aims to explore the use of a CNN combined with Dense layers and the raw wrist-worn accelerometry data to improve the classification of functional and non-functional UE movements in the paretic arm of stroke survivors. The functional category encompassed actions, e.g., gesturing, reaching and grasping objects, pushing to open a door, etc. The non-functional category included arm movements related to gait, sit-to-stand transitions, or whole-body movements that did not involve functional arm movement. Additionally, frames with no movement were also labeled as non-functional. By leveraging advanced deep learning techniques, this study seeks to enhance the classification performance of UE movements, offering a more objective approach to assessing rehabilitation outcomes. The goal is to improve the effectiveness of UE rehabilitation by providing better tools for monitoring and analyzing patient progress in both clinical and community settings.

## 2 Materials and methods

### 2.1 Data collection and preprocessing

In this study, 37 stroke survivors participated in a set of activities known as the Activity Script, which simulated daily tasks to reflect real-life upper extremity (UE) use in a community environment. Table 1 summarizes demographic and clinical characteristics of the 37 stroke survivors who participated in this study. Two subjects were excluded: subject 18 due to having only non-functional movements, and subject 24 due to corrupted data. Participants (24 males, 11 females) had a mean age of 59.4 ± 12.5 years (range: 32–84 years) and a median chronicity post-stroke of 16 months (range: 6–257 months). The sample included 18 individuals with left-sided and 17 with right-sided affected limbs. Arm impairment, assessed via the ARAT test, varied considerably across subjects (mean = 26.9 ± 15.1, range: 0–54). The mean use ratio, representing real-world affected-arm usage relative to the less-affected arm, was 0.5 ± 0.3 (range: 0–1.05). The broad variability in impairment severity and arm usage captured within this cohort provides context for interpreting model performance and evaluating the generalizability of our findings. We complied with all relevant ethical guidelines for human research. The study protocol was approved by the Institutional Review Board (IRB) at our institution. Informed consent was obtained from all participants prior to data collection.

**Table 1:** Summary of subject demographics including age, gender, and stroke information.

ID	Age (years)	Gender	Post (months)	Affected Limb	ARAT	Use Ratio
1	77	Male	23	Right	41	0.704
2	35	Male	35	Left	23	0.662
3	56	Male	17	Left	19	0.086
4	49	Female	19	Left	20	0.340
5	57	Male	104	Right	16	0.089
6	63	Male	77	Right	32	0.632
7	47	Female	12	Right	33	0.411
8	50	Male	53	Right	15	0.172
9	66	Male	69	Right	5	0.218
10	65	Male	20	Right	42	0.424
11	54	Female	24	Right	23	0.494
12	56	Female	26	Left	38	0.944
13	64	Male	14	Right	42	1.053
14	84	Female	6	Left	48	0.991
15	48	Male	9	Left	47	0.913
16	71	Male	15	Right	53	0.781
17	65	Male	17	Left	27	0.470
18	58	Male	17	Left	32	0.629
19	79	Male	18	Left	52	0.952
20	54	Male	12	Right	29	0.632
21	64	Male	11	Right	32	0.631
22	50	Male	36	Right	5	0.037
23	77	Male	10	Left	54	0.900
24	64	Female	7	Left	24	0.662
25	32	Female	6	Left	6	0.088
26	77	Female	13	Right	7	0.601
27	64	Male	8	Right	36	0.571
28	50	Female	257	Left	0	0.000
29	59	Male	13	Left	28	0.551
30	66	Male	14	Left	12	0.251
31	68	Female	14	Left	34	0.728
32	58	Male	16	Right	23	0.301
33	55	Male	14	Right	29	0.716
34	62	Female	13	Left	20	0.521
35	41	Male	10	Left	15	0.162
36	74	Male	137	Left	N/A	0.382
37	43	Female	29	Left	9	0.237

The data collection was conducted in a naturalistic environment within an Independence Square facility at MedStar National Rehabilitation Hospital. The setting included essential areas like a kitchen, bedroom, a shopping store, and a car. Participants performed various instrumental activities of daily living (IADLs) such as doing laundry, kitchen activities, grocery shopping, and bed making. In the laundry activity, participants moved clothes from a closet to a washer, transferred them to a dryer, and then folded or hung the clothes on hooks in the closet. In the kitchen activity, participants loaded and unloaded the dishwasher, cut an apple, picked up items from the floor, and used a broom to sweep the floor. In the shopping activity, participants gathered grocery items from a store, placed them in the car, and removed them from the car. In the bed-making activity, participants replaced the sheets and pillowcases on a bed.

**Figure 1:**
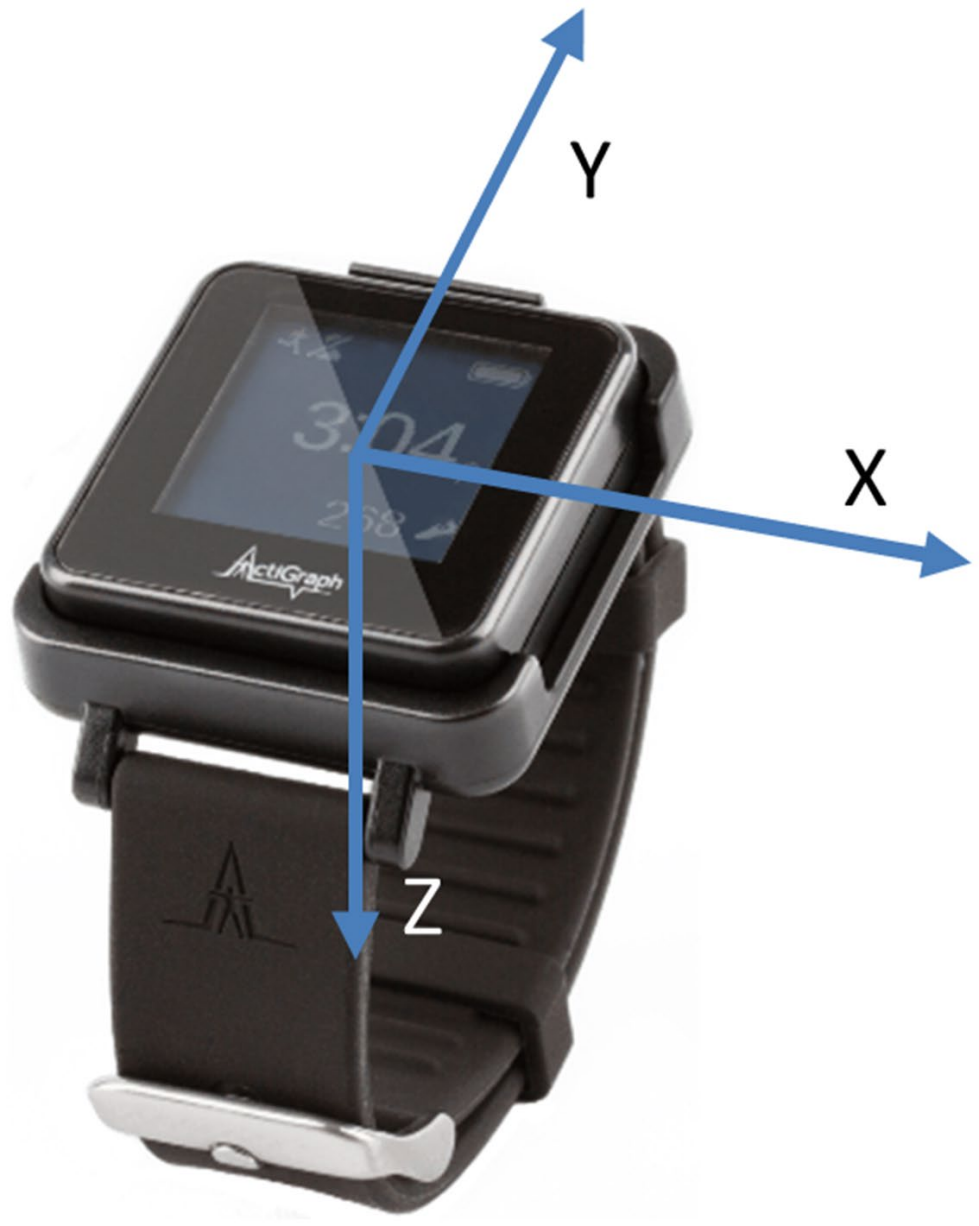
Picture of ActiGraph sensors with accelerometry axes labeled.

Participants performed these IADLs naturally, without specific instructions on which arm to use or whether to prioritize the paretic arm. They were instructed to perform the task as they usually would in the home and community, with activities interspersed with breaks, conversations with experimenters, and walking around the facility, allowing the collection of non-functional UE usage data. There was no strict time limit for completing the tasks. Table 2 summarizes the tasks performed by subjects, along with the minimum, maximum, and average duration of each task. We conducted data collection in two phases. In the first phase, 10 subjects were involved, and each subject performed only 4 tasks: doing laundry, kitchen activities, grocery shopping, and bed making. In the second phase, an additional 25 subjects were recruited, and each subject performed 7 tasks, with 3 additional tasks: letter writing, medication organizing, and keyboard typing, as clinicians felt that more seated tasks requiring hand dexterity should be added after the first phase. Throughout the experiments, participants wore IMU sensors on both wrists, similar to wrist watches or smart watches, and were videotaped. Figure 1 shows a picture of the ActiGraph sensor illustrating the axes directions.

**Table 2:** Tasks performed by subjects and duration statistics (in minutes).

# of Subjects	Doing laundry	Kitchen activities	Grocery shopping	Bed making	Letter writing	Medication organizing	Keyboard typing
10	x	x	x	x			
25	x	x	x	x	x	x	x
Min.	2.25	0.5	1.5	2	1	1.5	1.5
Max.	19.25	14.5	12	13	6	4.5	9.5
Mean	5.45	5.48	3.52	5.16	3.09	2.67	4.45

In the 2 phases of data collection, sensor data from the first 10 subjects were collected at 200 Hz with a commercially available sensor (ADIS16400BMLZ, Analog Devices) (Lum et al., 2020) while the remaining data were collected at 30 Hz or 50 Hz using the Actigraph GT9X Link watches. All raw accelerometry data were down-sampled to 30Hz before analysis and expressed as three-axis accelerations normalized to gravity (9.81 m/s^2^); no gravity compensation was applied. To ensure maximum generalizability, most external datasets include only raw accelerometry. In prior work, we also found no increase in model accuracy when using both accelerometry and angular velocity (Bochniewicz et al., 2017) compared to using accelerometry alone (Lum et al., 2020). The algorithms will operate with any wrist-worn three-axis accelerometer, provided that axis directions, sign conventions, and data format match those of the ActiGraph Link sensor; left and right arm data are made anatomically consistent with a simple axis transform, and a fixed 30 Hz sample rate is assumed. All these details will be provided as metadata in the data repository.

Three independent annotators reviewed the videos and labeled each frame as functional, non-functional, or unknown. Each limb was annotated independently, and the final label for each limb on each video frame was determined by majority vote. The agreement between annotators was quantified by the percentage of frames on which 2 independent annotators agreed on the class label. Across all possible comparisons and all limbs annotated, the mean agreement was 92.4 ± 5.3%. Most discrepancies are related to the precise timing of transitions in the class label. The video was synchronized with accelerometry data, and the ground truth labels for functional, non-functional, and unknown activity were transferred to the accelerometry data. Synchronization was achieved by rapidly oscillating the accelerometers in the z direction five times before attaching them to the subject. This produced five distinct peaks in the z-axis data, which were easily identified and marked. These sensor peaks corresponded to reversal points in the oscillation, which were marked on the video.

Frames labeled as “unknown” were initially used by human annotators for instances where the arm was out of view or when it was unclear whether the movement was functional or non-functional. However, these frames were later excluded before the training, leaving the ground truth with only two labels: “functional” and “non-functional.” After synchronization with the accelerometry data, the corresponding accelerometry points associated with the “unknown” labels were also removed. Then, the ground truth with 2 labels was used along with the raw accelerometry data for training an integrated deep learning model, specifically a Convolutional Neural Network (CNN) with Dense layers.

Using a custom Python IDE, we organized the collected sensor data in three different ways:

1. Paretic arm dataset: Comprising x_1_, y_1_, z_1_ dimensions using paretic arm labels
2. Non-paretic arm dataset: Comprising x_2_, y_2_, z_2_ dimensions using non-paretic arm labels
3. Combined arms dataset: Merging both arms’ data (x_1_, y_1_, z_1_, x_2_, y_2_, z_2_ dimensions) using paretic arm labels

The combined dataset incorporates data from both arms to capture a more comprehensive range of movement patterns. We then defined each data point as a 2-s sequence of sensor data. This approach yielded the following datasets:

1. Paretic arm dataset: 13,967 functional and 19,168 non-functional data points
2. Non-paretic arm dataset: 28,105 functional and 5,224 non-functional data points
3. Combined dataset: 13,923 functional and 19,075 non-functional data points

To maintain consistency, we excluded any data that did not conform to the 2-s sequence structure from our analysis. This method potentially truncates incomplete segments at the end of the sequence, which explains the slight variations in the final sample counts while preserving the time-series structure of the data, as seen in the paretic arm dataset and the combined dataset.

### 2.2 Network architecture

**Figure 2:**
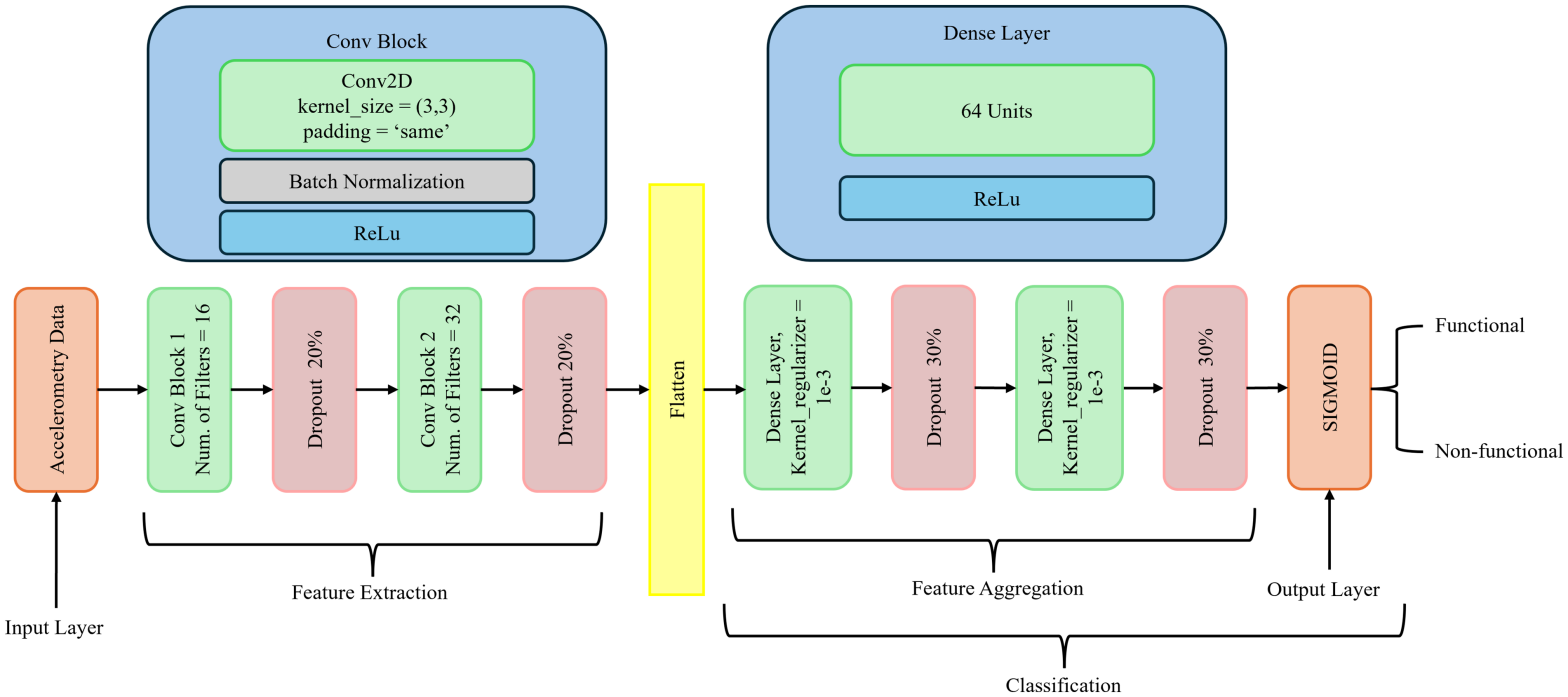
The proposed neural network architecture for classifying arm movements.

As depicted in Figure 2, our deep learning model utilizes CNNs with dense layers to classify both functional and non-functional arm movements using pre-processed raw accelerometry data. CNNs are specialized deep neural networks that process and analyze different data types. They are highly effective in image recognition and computer vision tasks due to their ability to capture spatial hierarchies. The key components of CNNs include convolutional layers that apply filters to produce feature maps, activation functions such as ReLU to introduce non-linearity, pooling layers to downsample feature maps, dense layers for high-level reasoning, and dropout for regularization. These elements enable CNNs to efficiently learn and detect various features within images, reducing the number of parameters through parameter sharing and capturing spatial dependencies through local receptive fields.

While accelerometry data is fundamentally temporal, CNN-based models have demonstrated effectiveness in capturing local temporal–spatial patterns and hierarchical features within fixed-length accelerometry segments, making them particularly suitable for our classification tasks. Prior research comparing neural architectures for accelerometry-based human activity recognition (HAR) has shown that CNNs often achieve superior or comparable accuracy relative to recurrent neural networks (RNNs) such as LSTMs, while requiring less training time and fewer computational resources (Hammerla et al., 2016; Ignatov, 2018). LSTM-based models, though excellent at modeling long-range temporal dependencies, are generally more computationally demanding and prone to overfitting with relatively smaller or fixed-length datasets typical in stroke rehabilitation research. Preliminary experiments conducted during the early stages of our study showed that CNN architecture outperformed basic LSTM models in terms of validation accuracy and computational efficiency for short-duration (e.g., 2-s) accelerometry windows, consistent with findings in the literature (Bochniewicz et al., 2017; Lum et al., 2020). Transformer-based models, although powerful for processing sequential data, typically require extensive training data to prevent overfitting and fully demonstrate their potential (Vaswani et al., 2017). Given the limited size and diversity of available stroke rehabilitation data, CNN architectures combined with dense layers offer a robust and computationally efficient alternative, capable of capturing essential local and global features without the high data demands associated with transformer-based approaches.

The proposed architecture is designed to capture both local and global features from the data. CNN excels at extracting local features, spatial, and temporal hierarchies, while Dense layers integrate and map these features to the final output, enabling the modeling of complex global patterns and decision boundaries. This combination enhances the model’s ability to learn detailed and hierarchical features, improving its accuracy and robustness in classifying activities. The integration of CNN with Dense layers improves both classification accuracy and training efficiency, particularly in distinguishing between non-functional (NF) and functional (F) movements of the paretic arm. Dense layers play a critical role in consolidating the extracted features and mapping them to output classes, enabling the network to model complex relationships and decision boundaries. By leveraging both local feature extraction and global pattern integration, the architecture becomes more robust and less prone to overfitting. This design is especially well-suited for applications such as human activity recognition, gesture recognition, and other domains where precise activity classification is critical. Thus, it provides a powerful framework for developing models that can accurately and efficiently classify various activities from complex data inputs.

Batch normalization, kernel regularization with l2, and dropout are used between layers to prevent overfitting and stabilize the training process. Batch normalization normalizes each layer’s inputs by adjusting and scaling the activations, reducing internal covariate shifts, and improving gradient flow. Dropout randomly sets a fraction of the input units to zero during training, preventing the network from becoming too reliant on specific neurons and ensuring better generalization.

To evaluate the influence of three data configurations, we trained and built three models:

1. Paretic Arm Model: The proposed network was trained solely with paretic arm data
2. Non-Paretic Arm Model: The proposed network was trained solely with non-paretic arm data
3. Combined Arms Model: The proposed network was trained with merged data from both arms

These experimental configurations allow us to compare models’ performance across different data scenarios and assess the potential benefits of incorporating data from both arms in the analysis.

Figure 2 illustrates the proposed neural network architecture for classifying arm movements from accelerometry data. The network is structured into three main stages: feature extraction, feature aggregation, and classification. In the feature extraction stage, the input consists of preprocessed accelerometry data. It is passed through two Conv2D blocks. The first convolutional block employs 16 filters with a 3 × 3 kernel size, followed by batch normalization, ReLU activation, and a 20% dropout layer to mitigate overfitting. The second block is similar but uses 32 filters, again followed by batch normalization, ReLU activation, and another 20% dropout. These convolutional layers are designed to capture local spatial and temporal patterns in the accelerometry signals. The feature aggregation stage begins by flattening the output of the last convolutional layer. This is followed by two Dense layers, each containing 64 units. Both dense layers use ReLU activation, kernel L2 regularization (*λ* = 1e-3) to prevent overfitting, and are followed by dropout layers with a 30% dropout rate. These layers enable the learning of higher-level feature representations from the extracted signal patterns. The final classification stage consists of a single layer with a sigmoid activation function that outputs a probability value representing the likelihood of the movement being functional or non-functional. The model was trained using a batch size of 64 for up to 700 epochs. A learning rate scheduler was applied to reduce the learning rate by a factor of 0.1 if the validation loss did not improve for 10 consecutive epochs. Early stopping was also used, with a patience of 20 epochs, to halt training if no improvement was observed, thereby preventing overfitting. Detailed hyperparameter settings are listed in Table 3.

**Table 3:** Hyperparameters used in model training.

Parameter name	Batch size	Kernel	Epochs	Learning rate	Early stopping
Val	64	Activation(“Relu”) L2(1e-3)	700	Adam (1e-3) Factor = 0.1 Patience = 10	Patience = 20

### 2.3 Model implementation and evaluation

For implementation, we utilized the TensorFlow2 and Keras frameworks (Bisong, 2019). TensorFlow2 is an open-source deep learning library developed by Google that provides a flexible and comprehensive ecosystem for building and deploying deep learning models. Keras is a high-level API for neural networks, written in Python and capable of running on top of TensorFlow, that simplifies the process of building and training deep learning models. The accelerometry data were normalized using RobustScaler from Scikit-learn (Pedregosa et al., 2011), which is particularly effective in handling outliers by removing the median and scaling the data according to the interquartile range, thus making it robust to anomalies. Robust Scaler equation:


$$X_{\mathrm{scaled}}=\frac{X-Q2}{\mathrm{IQR}}$$

where:

- X is the original data point
- Q2 is the median of the data
- IQR is the interquartile range, calculated as Q3 − Q1, where Q1 is the first quartile (25th percentile) and Q3 is the third quartile (75th percentile).

Thus, the transformed data X_scaled_ has its median centered around zero and is scaled according to the interquartile range, which helps mitigate the influence of outliers.

Performance evaluation of the intrasubject model, i.e., within-subject model, was conducted using 5-fold cross-validation. In this process, the data for each subject was divided into five equal parts, or “folds.” The model was trained on four of the five folds, with the remaining fold serving as the test set. This process was repeated five times, using a different fold as the test set, ensuring that every part of the data was used for both training and testing. The results from the five iterations were then averaged to provide a robust assessment of the model’s performance for each subject.

In contrast, performance evaluation of the intersubject model, i.e., across-subject model, was conducted using leave-one-out cross-validation, where one subject was left out as the test set while the model was trained on the remaining subjects. This process was repeated for each subject, thoroughly assessing the model’s performance across different subjects.

Additionally, we employed ReduceLROnPlateau, a callback in Keras that monitors quantity and reduces the learning rate by a factor of 0.1 if no improvement is seen for a ‘patience’ number of epochs, starting from 1e-3 and adjusting every 10 epochs. Early stopping was used to prevent overfitting by monitoring validation accuracy and stopping the training process once performance ceased to improve for every 20 epochs, preserving the best weights encountered during training. This comprehensive approach was adopted to ensure robust model training and accurate assessment of functional and non-functional movements in stroke rehabilitation.

The model was built and trained on Paperspace CloudServer. This study evaluated model performance using two primary metrics: accuracy and F1 scores for classifying non-functional and functional arm movements. Accuracy measures the overall correctness of the model’s predictions, representing the proportion of correct predictions (true positives and true negatives) among all predictions. F1 scores offer a balanced assessment by considering both precision (the accuracy of positive predictions) and recall (the ability to identify all positive instances correctly), which is particularly valuable in subjects with low functional use.

## 3 Results

Tables 4, 5 report the model performance metrics as mean ± standard deviation, conveying both the central tendency and the variability in the model’s performance across subjects. Table 4 summarizes several performance metrics for the intrasubject model trained with the paretic arm, non-paretic arm dataset, and combined arms datasets. Note that subject 28, who exhibited no functional use, was excluded from the intrasubject modeling but included in the intersubject modeling.

**Table 4:** Comparison of intrasubject model performance metrics for paretic arm, non-paretic arm, and combined arms.

Model	Training accuracy (±std)	Validation accuracy (±std)	Training F1 score for NF class (±std)	Validation F1 score for NF class (±std)	Training F1 score for F class (±std)	Validation F1 score for F class (±std)
RF – Paretic arm	1.00 ± 0.00	0.91 ± 0.04	1.00 ± 0.00	0.90 ± 0.06	1.00 ± 0.00	0.81 ± 0.19
RF – Non-paretic arm	0.99 ± 0.02	0.96 ± 0.06	0.99 ± 0.03	0.83 ± 0.12	0.99 ± 0.02	0.97 ± 0.04
RF – Combined arms	1.00 ± 0.00	0.92 ± 0.03	1.00 ± 0.00	0.90 ± 0.06	1.00 ± 0.00	0.83 ± 0.19
Paretic arm	0.96 ± 0.03	0.90 ± 0.05	0.95 ± 0.05	0.89 ± 0.08	0.90 ± 0.15	0.81 ± 0.18
Non-paretic arm	0.97 ± 0.02	0.96 ± 0.04	0.90 ± 0.11	0.85 ± 0.13	0.98 ± 0.02	0.97 ± 0.03
Combined arms	0.98 ± 0.02	0.91 ± 0.04	0.97 ± 0.03	0.90 ± 0.06	0.94 ± 0.11	0.84 ± 0.14

**Table 5:** Comparison of intersubject model performance metrics for paretic arm, non-paretic arm, and combined arms.

Model	Training Accuracy (±std)	Validation Accuracy (±std)	Training F1 score for NF class (±std)	Validation F1 score for NF class (±std)	Training F1 score for F class (±std)	Validation F1 score for F class (±std)
RF – Paretic arm	1.00 ± 0.00	0.68 ± 0.12	1.00 ± 0.00	0.67 ± 0.16	1.00 ± 0.00	0.58 ± 0.20
RF – Non-paretic arm	0.99 ± 0.00	0.89 ± 0.07	0.98 ± 0.00	0.57 ± 0.20	0.99 ± 0.00	0.93 ± 0.05
RF – Combined arms	1.00 ± 0.00	0.72 ± 0.10	1.00 ± 0.00	0.72 ± 0.16	1.00 ± 0.00	0.61 ± 0.19
Paretic arm	0.79 ± 0.03	0.79 ± 0.06	0.80 ± 0.06	0.74 ± 0.16	0.73 ± 0.07	0.67 ± 0.18
Non-paretic arm	0.93 ± 0.03	0.89 ± 0.04	0.70 ± 0.15	0.55 ± 0.23	0.96 ± 0.02	0.94 ± 0.03
Combined arms	0.91 ± 0.02	0.88 ± 0.10	0.86 ± 0.04	0.63 ± 0.35	0.92 ± 0.03	0.88 ± 0.15

The intrasubject model performs well for the paretic arm, with a training accuracy of 0.96 ± 0.03 and a validation accuracy of 0.90 ± 0.05. For non-functional movement detection, the validation F1 score is 0.89 ± 0.08, while functional movement detection yields a lower F1 score of 0.81 ± 0.18. The increased variability in functional movement F1 scores is primarily attributed to three stroke survivors with more severe impairments, leading to imbalanced datasets where less than 10% of movements were functional. Specifically, subjects 3, 22, and 25 had functional use of 5.8, 3, and 7.6%, respectively. Their validation functional movement F1 scores were 0.19 ± 0.12, 0.52 ± 0.34, and 0.20 ± 0.27, respectively.

Several strategies can mitigate this imbalance, including upsampling, synthetic minority oversampling (SMOTE; Chawla et al., 2002), and class weighting. Table 6 illustrates performance improvements using SMOTE, where the minority class size is set to 50% of the majority class, resulting in a functional use increase to 33.3%. After applying SMOTE, the validation F1 scores for these subjects improved to 0.32 ± 0.08, 0.61 ± 0.39, and 0.28 ± 0.38, respectively. While SMOTE enhances results compared to the original data, the limited functional data still constrains the model’s generalizability and stability. Removing these three subjects from our analysis improves the functional movement F1 score to 0.94 ± 0.05 for training and 0.85 ± 0.10 for validation. Model performance also improves for the combined arms dataset in both training and validation. Non-functional movement detection achieved a validation F1 score of 0.90 ± 0.06, whereas functional movement detection showed more variability, with a validation F1 score of 0.84 ± 0.14.

**Table 6:** Effect of SMOTE on intrasubject model performance for paretic and combined arm models for three imbalance subjects.

Model	Subject #	Functional use	Original data			with SMOTE (F/NF = 0.5)		
			Accuracy	F-Score (F)	F-Score (NF)	Accuracy	F-Score (F)	F-Score (NF)
Paretic arm	3	0.058	0.95 ± 0.00	0.19 ± 0.12	0.97 ± 0.01	0.94 ± 0.01	0.32 ± 0.08	0.96 ± 0.01
	22	0.030	0.98 ± 0.01	0.52 ± 0.34	0.99 ± 0.00	0.99 ± 0.01	0.61 ± 0.39	0.99 ± 0.01
	25	0.076	0.96 ± 0.01	0.20 ± 0.27	0.98 ± 0.00	0.97 ± 0.01	0.28 ± 0.38	0.98 ± 0.01
Combined arms	3	0.058	0.96 ± 0.01	0.41 ± 0.28	0.98 ± 0.01	0.95 ± 0.01	0.46 ± 0.19	0.98 ± 0.01
	22	0.030	0.98 ± 0.01	0.57 ± 0.22	0.99 ± 0.00	0.98 ± 0.01	0.57 ± 0.22	0.99 ± 0.00
	25	0.076	0.97 ± 0.01	0.25 ± 0.25	0.98 ± 0.00	0.97 ± 0.01	0.35 ± 0.36	0.98 ± 0.00

Compared to the RF baseline, as shown in Table 4, the proposed model exhibits similar validation accuracy for the paretic arm (0.90 ± 0.05 vs. 0.91 ± 0.04) and non-paretic arm (0.96 ± 0.04 vs. 0.96 ± 0.06) but shows improved validation F1 scores for functional movements in the combined dataset (0.84 ± 0.14 vs. 0.83 ± 0.19). Although CNN improvement is not significant, the CNN model operates directly on raw accelerometer data, bypassing the need for handcrafted features required by RF models, which may be advantageous with larger datasets.

Table 5 presents the same set of performance metrics for the intersubject model among three datasets: paretic arm, non-paretic arm, and combined arms. Across all datasets, the intersubject models show a common trend where training accuracy and F1 scores are higher than their validation counterparts. The model trained on the paretic arm data performs consistently with training and validation accuracies of 0.79 ± 0.03 and 0.79 ± 0.06, respectively. However, it shows lower F1 scores for functional movements, particularly in validation, with a score of 0.67 ± 0.18, indicating some variability in classifying paretic arm movements. The non-paretic arm model demonstrates consistently higher accuracy and F1 scores, particularly for functional movements. It achieves a training accuracy of 0.93 ± 0.03 and a validation accuracy of 0.89 ± 0.04. It excels in classifying functional movements, with high F1 scores in both training at 0.96 ± 0.02 and validation at 0.94 ± 0.03 but shows lower performance for non-functional movements. These results reflect the predictability and consistency of movement patterns in the non-paretic arm, though the lower F1 scores for non-functional movements indicate greater challenges in capturing these patterns. The combined model, which incorporates data from both arms, performs well overall, achieving a training accuracy of 0.91 ± 0.02 and a validation accuracy of 0.88 ± 0.10, respectively. This model maintains high F1 scores for functional movements while improving on the classification of non-functional movements compared to the non-paretic arm model alone. These results suggest that incorporating bilateral data provides a richer feature set, enabling the model to generalize more effectively across different movement types, though the broader variability in validation scores underscores the complexity of generalizing across subjects.

Compared to RF, the proposed CNN model achieved substantially higher validation F1 scores for functional movements in the paretic arm (0.67 ± 0.18 vs. 0.58 ± 0.20), non-paretic arm (0.94 ± 0.03 vs. 0.93 ± 0.05), and especially the combined arms dataset (0.88 ± 0.15 vs. 0.61 ± 0.19).

These results underscore the combined arms model’s capacity to leverage richer bilateral features, providing enhanced robustness and improved generalization. However, maintaining balanced datasets remains crucial for achieving consistently high performance across all subjects.

**Figure 3:**
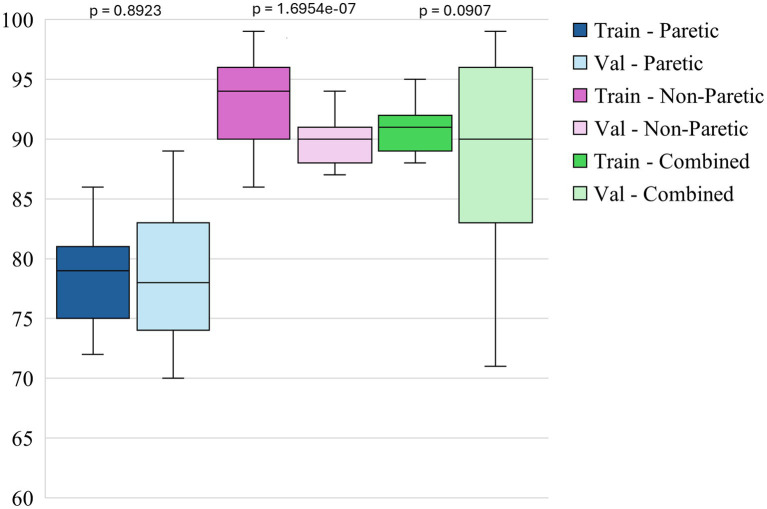
Intersubject model- training and validation accuracies across datasets.

**Figure 4:**
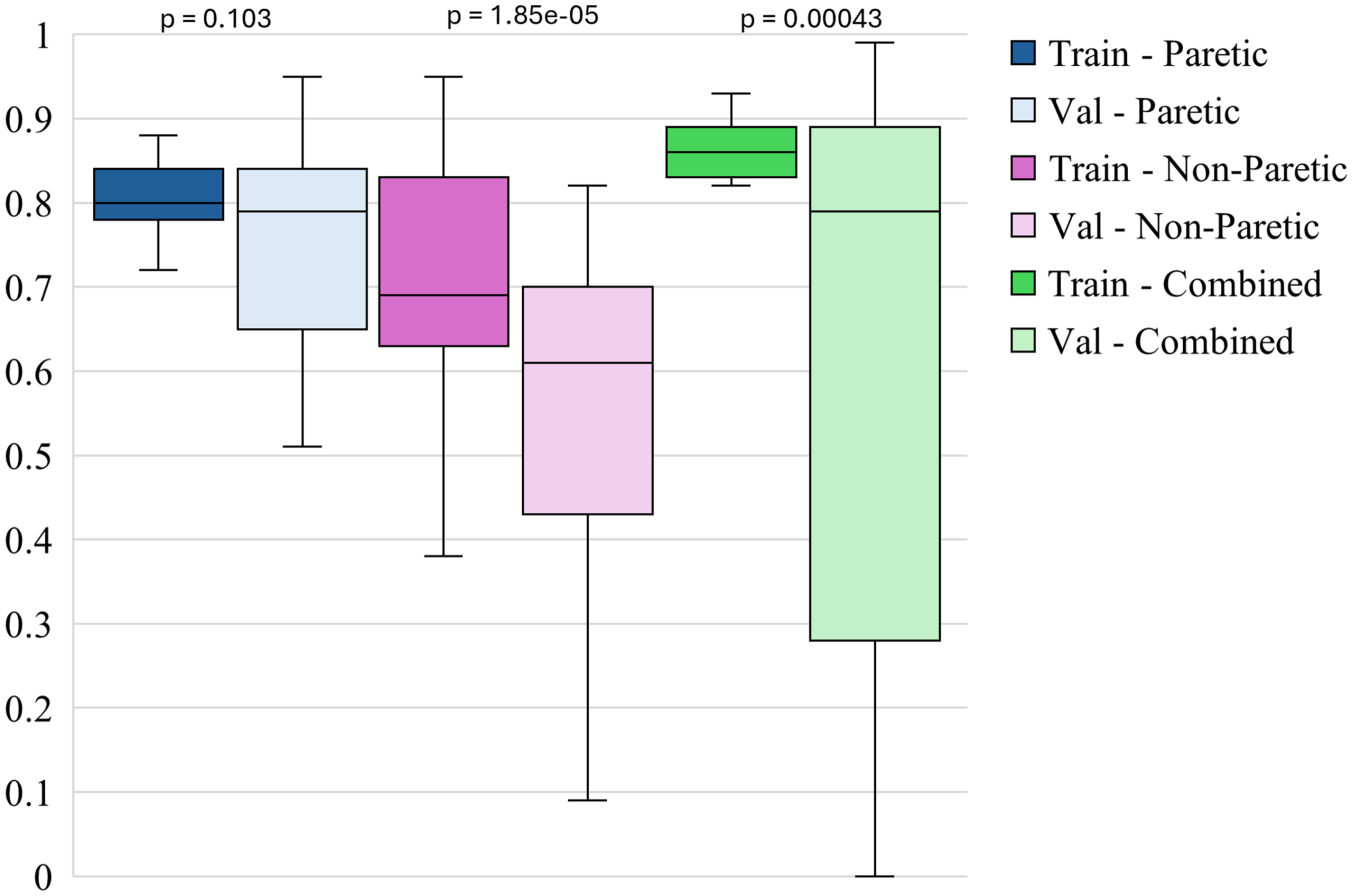
Intersubject model- training and validation f1 score non- functional across datasets.

**Figure 5:**
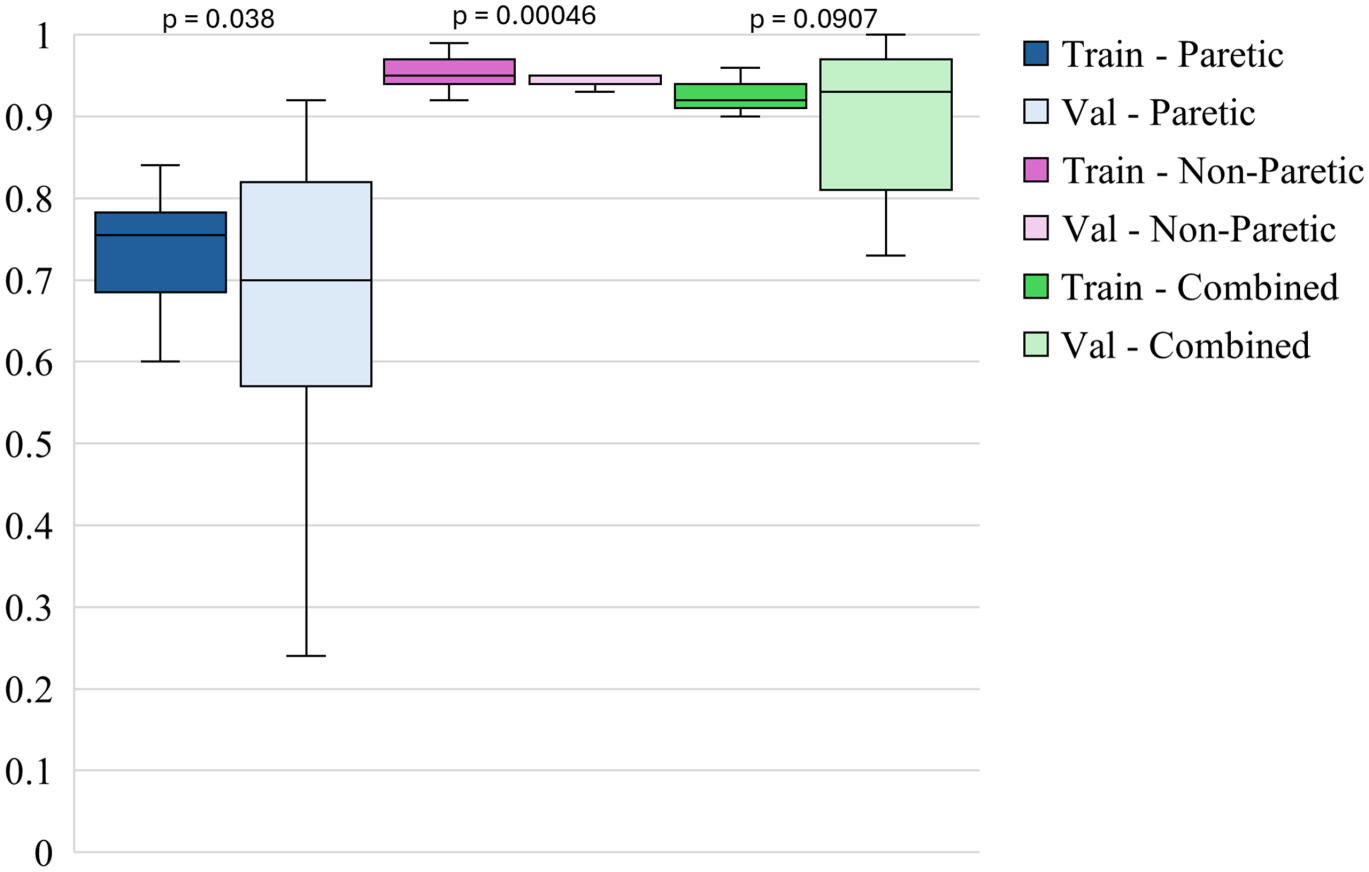
Intersubject model- training and validation f1 score functional across datasets.

The box plots in Figures 3–5 illustrate three different statistical and performance metrics based on three different training datasets: the paretic arm, the non-paretic arm, and the combined arms dataset.

Figure 3 shows the box plots of model accuracy for 3 different datasets. The non-paretic arm model consistently performs better in both the training and validation phases than the other 2 models. The median training accuracy reaches 94%, while the validation accuracy is approximately 90%. This high level of performance with small variability suggests that the non-paretic arm movement is more predictable and has consistent movement patterns, making it easier for the model to learn from. Additionally, the smaller range of variability in training and validation accuracies indicates that the model performs reliably and robustly. The combined dataset, which includes data from both the paretic and non-paretic arms, also shows strong results. The median training accuracy is around 91%, while the validation accuracy is about 90%. The combined data from both arms seems to provide a richer and more comprehensive dataset, enabling the model to generalize well across different types of movements. The slightly broader range of validation accuracies in this dataset suggests that while the model is robust, it benefits from the diversity of data, enhancing its ability to handle variations in movement patterns from both arms. This suggests that leveraging bilateral data can be an effective approach for enhancing model accuracy and generalization. The paretic arm model, which represents movements from the paretic arm, exhibits median training and validation accuracies of approximately 79 and 78%, respectively. While slightly lower than the non-paretic and combined datasets, these accuracies still reflect solid model performance, especially given the inherent complexities and variability in the movement data from stroke-paretic limbs. The model’s ability to achieve these accuracies suggests it is effectively learning from the more challenging data, and the consistent performance across training and validation phases indicates that the model is generalizing well to unseen data.

Figure 4 shows the box plots of model F1 scores for non-functional movements for 3 different datasets. The non-paretic arm model consistently performs well during training, with a median F1 score of around 0.69 and a relatively narrow range, indicating stable performance across different training iterations. However, in the validation phase, the median F1 score drops to approximately 0.61 with a wider range of variability. This suggests that while the model can reliably identify non-functional movements during training, it struggles more during validation, potentially due to the more subtle or less distinct movement patterns of the non-paretic arm compared to the paretic arm. The combined model exhibits the highest performance overall in terms of F1 scores for non-functional movements. The training F1 score reaches a median of about 0.86, indicating that exposure to a diverse set of movement patterns enables the model to learn and classify non-functional movement more effectively. During validation, the combined model maintains a higher median F1 score of around 0.79, outperforming the individual models. This robustness suggests that leveraging bilateral data improves the model’s ability to generalize, making it more adaptable to different non-functional movement patterns. The paretic arm model exhibits solid performance, particularly in training, with a median F1 score of around 0.81. This outcome suggests that the model effectively learns from the more variable and complex data associated with the paretic arm’s movements. In the validation phase, the median F1 score remains consistent at approximately 0.79, though with a broader spread, indicating some variability. This variability suggests that, while the model generally performs well in detecting non-functional movements in the paretic arm, certain validation instances pose additional challenges. Overall, the paretic arm model provides a strong basis for training, but additional strategies may enhance its generalization in validation.

Figure 5 shows the box plots of model F1 scores for functional movements for 3 different datasets. The non-paretic arm model performs exceptionally in detecting functional movements during the training and validation phases. The training F1 scores for this model are consistently high, with a median of around 0.95 and minimal variability. This indicates that the model is highly effective at learning the pattern associated with functional movements in the non-paretic arm. During validation, the F1 scores remain robust, with a median of nearly 0.94, indicating strong generalization capabilities. The narrow range of these scores further underscores the model’s reliability in identifying functional movements when applied to non-paretic arm data. The combined model, which includes data from both the paretic and non-paretic arms, also demonstrates strong performance in detecting functional movements. The training F1 scores for the combined model have a median of around 0.92, suggesting that the model benefits from the richer and more varied feature set provided by integrating data from both arms. This improved performance carried over into the validation phases indicates that using bilateral data enhances the learning process, allowing the model to generalize effectively across varied conditions. The paretic arm model shows solid, though slightly lower, performance in detecting functional movements compared to the non-paretic arm and combined models. The training F1 scores for this model have a median of around 0.74, suggesting that, while the model effectively learns from the paretic arm data, the variability inherent in post-stroke movements poses certain challenges. During validation, the median F1 score drops slightly to around 0.70, with a broader spread of scores, which may reflect the increased complexity and variability of functional movement patterns in the paretic arm. Despite this, the model’s ability to maintain relatively high F1 scores indicates it can effectively classify functional movements, even in more challenging datasets.

Paired statistical comparisons between training and validation performance indicated significant discrepancies across datasets and metrics. For classification accuracy, the difference was not statistically significant for the paretic arm model (*p* = 0.8923) or the combined arms model (*p* = 0.0907) but was highly significant for the non-paretic arm model (*p* = 1.695 × 10^−7^). Regarding the F1 score for non-functional movements, no significant difference was observed for the paretic arm model (*p* = 0.103), whereas significant differences were found for the non-paretic (*p* = 1.85 × 10^−5^) and combined arms models (*p* = 0.00043). In contrast, the F1 score for functional movements revealed significant differences for the paretic (*p* = 0.038) and non-paretic arm models (*p* = 0.00046), while the combined arms model did not show a statistically significant difference (*p* = 0.097). These findings suggest that models trained on non-paretic arm data may be more susceptible to overfitting, as evidenced by substantial performance drops from training to validation, while models trained on paretic and combined arms data exhibited more stable generalization.

Overall, the figures highlight the relative strengths of each dataset in training and validating models for classifying arm movements. The analysis of performance metrics reveals that the non-paretic arm dataset consistently provides higher accuracy and F1 scores, reflecting the stability and predictability of the movement patterns it represents. The combined dataset, which merges data from both arms, also performs exceptionally well, underscoring the value of using a more comprehensive dataset that captures a wider range of movement characteristics. Adding these features significantly enhances the model’s overall effectiveness, especially in training, and improves generalization, though some variability remains. While presenting more of a challenge, the paretic arm dataset still results in strong model performance, demonstrating the model’s capability to learn and generalize from more complex and variable data. These findings underscore the importance of leveraging comprehensive datasets and incorporating diverse features to optimize model robustness and accuracy, particularly in the context of complex movement classification tasks in stroke rehabilitation.

Table 7 reports the effect sizes (Cohen’s d) and corresponding 95% confidence intervals for intersubject comparisons across three training datasets: paretic arm, non-paretic arm, and combined arms. The combined dataset, which integrates signals from both arms, yielded moderate effect sizes across accuracy and F1 metrics, reflecting more balanced generalization compared to the individual datasets. The paretic arm dataset showed negligible differences between training and validation performance (e.g., accuracy *d* = −0.025), suggesting consistent generalization but limited variability in movement patterns. By contrast, the non-paretic dataset exhibited large effect sizes for accuracy (*d* = 1.162) and F1 scores, indicating strong training performance but a marked drop during validation, consistent with overfitting. The combined dataset produced intermediate results, with reduced overfitting compared to the non-paretic case and improved stability relative to the paretic case. These findings indicate that leveraging both paretic and non-paretic signals can capture a wider spectrum of movement characteristics, supporting more robust and generalizable classification of upper extremity function.

**Table 7:** Comparison of intersubject statistical metrics for paretic arm, non-paretic arm, and combined arms.

Model comparison	Metrics	Cohen’s d	95% confidence intervals
Train – Paretic vs. Val – Paretic	Accuracy	−0.025	[−0.502, 0.452]
	F1 Functional	0.429	[−0.053, 0.912]
	F1 Non-functional	0.349	[−0.131, 0.830]
Train – Non-Paretic vs. Val – Non-Paretic	Accuracy	1.162	[0.647, 1.678]
	F1 Functional	0.809	[0.313, 1.305]
	F1 Non-functional	0.760	[0.266, 1.254]
Train – Combined vs. Val – Combined	Accuracy	0.410	[−0.072, 0.892]
	F1 Functional	0.415	[−0.067, 0.897]
	F1 Non-functional	0.908	[0.407, 1.409]

## 4 Discussion

### 4.1 Interpretation of results

The intrasubject models perform very well when trained with the combined arms data. The paretic arm model shows a higher variability in performance due to data imbalance and subject-specific movement patterns. Note that the accuracy and F1 scores were improved if the three subjects with imbalanced data were removed.

The intersubject model demonstrated improvement in accuracy and F1 scores across different datasets using raw accelerometry data. The trained model using the paretic arm data achieved a training accuracy of 79% and a validation accuracy of 79%. These results show an improvement over the previously reported validation accuracy of 74.2% using the paretic arm data by (Lum et al., 2020). Achieving this level of accuracy is encouraging, given the inherent variability and complexity associated with movement data from stroke-paretic limbs, as well as the limited training data available. In comparison, the model trained on the non-paretic arm displayed higher performance, with a validation accuracy of 89%, suggesting that the more predictable and consistent movement patterns of the non-paretic arm are easier for the model to learn and generalize from. The combined arms model significantly improved the performance of the paretic arm model. With this integrated dataset, the model achieved a validation accuracy of 88%, surpassing the accuracy of the model trained solely on the paretic arm data. The F1 scores for non-functional movements in the combined dataset improved to 0.86 during training, compared to 0.80 for the paretic arm alone, highlighting the benefits of a richer feature set. However, the validation F1 score for non-functional movements showed greater variability, indicating that while the additional data enhances the model’s learning capacity, it also introduces complexity that may complicate generalization. The combined dataset maintained strong validation F1 scores of 0.88 for functional movements, demonstrating the model’s effective generalization across various movement conditions.

These results also align with the broader trends observed in machine learning applications within healthcare (Figueroa et al., 2012; Beam and Kohane, 2018; Li et al., 2023; Nunes et al., 2024) where models often perform better with more consistent and less variable data. The stable movement patterns of the non-paretic arm provided a more stable foundation for training, which likely contributed to the model’s enhanced performance in both the training and validation phases (Hossain et al., 2023).

Additionally, this study included a larger sample of 35 subjects across a wider range of impairment severities and incorporated three more tasks per subject for the 25 newly recruited subjects compared to the 10 subjects in (Lum et al., 2020). This larger, more diverse dataset may have also contributed to our model’s higher validation accuracies for both the paretic arm and combined arms models, underscoring its improved generalizability.

In addition, the training duration, measured by the number of epochs needed to reach the optimal performance, is shorter for the non-paretic arm model (e.g., 50–75 epochs) than for the paretic arm model (e.g., ~100 epochs). This faster convergence may suggest that the model learns more consistent or generalizable patterns from the non-paretic arm data, possibly due to reduced movement variability or imbalances in that dataset.

This study utilizes raw accelerometry signals as input, which enables the model to learn rich, hierarchical representations directly from the data; however, this approach results in more complex and less interpretable deep learning models. To enhance interpretability and transparency, future work will integrate advanced attribution methods such as saliency maps, feature importance analyses, integrated gradients, and related techniques. These approaches will be able to identify and visualize the temporal and spatial features that drive the classification of functional versus non-functional movements, thereby fostering clinical trust and supporting model validation and refinement.

In our current model training, hyperparameters were chosen through an iterative, empirical process informed by pilot experiments on a subset of data. Moving forward, we plan to adopt more sophisticated hyperparameter optimization techniques, such as Bayesian optimization, which efficiently balance exploration and exploitation of the hyperparameter space. This probabilistic approach builds a surrogate model of the objective function, guiding the search for optimal hyperparameters to improve model accuracy while minimizing computational cost. Integrating such automated optimization will further enhance training robustness, reduce overfitting, and accelerate convergence, especially as datasets grow in size and complexity.

### 4.2 Practical implications

The rationale for using raw accelerometry data lies in its ability to capture complex movement patterns, which are essential for accurately assessing UE movements. We gain a comprehensive source of information by utilizing raw data, free from subjective interpretations, such as patient self-reports or other clinical assessments. We obtained highly accurate and reliable classifications by processing and analyzing accelerometer data using advanced models like CNNs combined with Dense layers. This approach supports a more objective and quantifiable assessment of functional and non-functional movements, thereby enhancing the overall effectiveness of stroke rehabilitation monitoring.

The findings of this study have important implications for creating and developing personalized rehabilitation strategies. The high accuracy and stability of our models have the potential to enable more precise monitoring of UE functionality, allowing clinicians to tailor interventions based on individual performance metrics. In clinical practice, these models can be used to provide real-time feedback and facilitate long-term monitoring, thereby improving the quality of rehabilitation programs. The demonstrated robustness of the models also ensures reliability across different cases, which is critical for real-world applications where patient data can vary widely.

Although our architecture is relatively compact, we have not systematically benchmarked the computational efficiency and feasibility of deploying these models in real-time clinical or home environments. The model processes data in 2-s windows using a modest number of convolutional filters and dense layers, making it computationally efficient enough in principle for deployment on modern mobile or edge devices. However, real-time deployment on resource-constrained platforms such as wearable devices or smartphones requires explicit profiling to evaluate inference latency, energy consumption, and memory usage under real-world conditions. Prospective studies should also assess sensor runtime under different sampling/transmission schemes and develop strategies to maximize adherence, such as calibration routines, wear-time monitoring, and user feedback.

Despite the promising results, the study has limitations. Although the dataset (35 subjects) is larger than previous studies (10 subjects) and involves more tasks (7 tasks in total), it may still be insufficient and could restrict the generalizability of the models. Deploying deep learning models for upper extremity movement classification in real-world environments entails several challenges that may impact their reliability and generalizability. Sensor placement variability is one of the most critical issues, as small differences in the orientation and positioning of wrist-worn sensors can introduce significant signal inconsistencies and reduce model performance. To address this, data collection could incorporate a standardized calibration procedure, such as holding the arm in a standardized posture prior to data collection, aligning sensor axes, and normalizing orientation. Another important consideration is signal noise and artifacts, which can arise from environmental vibrations or electromagnetic interference. Incorporating real-time signal filtering, a robust preprocessing pipeline, and anomaly detection algorithms may help mitigate these effects and preserve data quality.

Furthermore, differences across patient populations present challenges for model generalization, as stroke survivors exhibit substantial variability in impairment severity, compensatory strategies, and daily activity patterns. To improve performance across diverse cohorts, future work should prioritize the collection of larger, multi-center datasets representing a broad spectrum of functional abilities and demographic characteristics. Finally, recognizing that activities performed in home and community settings are inherently more variable than scripted rehabilitation tasks, it will be essential to conduct prospective validation studies under ecologically valid conditions. Addressing these challenges systematically will be critical to developing reliable, scalable systems that can support continuous monitoring and personalized rehabilitation in real-world practice. Additionally, exploring other deep learning architectures and hyperparameter tuning could potentially improve performance. Incorporating multimodal data, such as clinical assessments, might also improve the model’s accuracy and robustness. Finally, the results of this study are not generalizable to acute stroke settings, as all subjects were more than 6 months post-stroke. Additionally, generalization to patient performance in the home and community will have to be demonstrated with data collection in these environments.

## 5 Conclusion

This study demonstrated that deep learning models, particularly those leveraging CNNs with Dense layers, can accurately and reliably classify functional and non-functional arm movements in stroke patients. The proposed models surpass previous benchmarks in performance.

This research contributes significantly to stroke rehabilitation by offering a robust and precise method for classifying arm movements using raw accelerometry data. The findings highlight the potential of advanced deep learning algorithms to enhance the monitoring and assessment of UE functionality, which is essential for developing personalized rehabilitation strategies.

We acknowledge key limitations in our study, including sensor placement variability, noise/artifacts, and limited sample size. Small variations in wrist sensor positioning can introduce significant signal inconsistencies that may reduce model performance. Additionally, the relatively small and heterogeneous dataset limits generalizability, especially across diverse patient populations and real-world home environments. Future work will focus on standardized calibration procedures and collecting larger multi-center datasets to improve model robustness and ecological validity.

Potential challenges related to sensor battery life, data transmission, and user compliance must be carefully considered in real-world deployments. Prospective studies should evaluate sensor runtime under various sampling rates and data transmission schemes to identify optimal configurations that balance performance and power consumption.

Strategies to maximize user adherence include implementing calibration routines to ensure data quality, wear-time monitoring to track device usage, and providing timely user feedback to encourage consistent wear. Advanced power management techniques—such as adaptive sampling, low-power modes, and efficient data communication protocols—can significantly extend battery life in wearable devices.

Future work should integrate these approaches and explore energy-harvesting technologies to sustain longer operational times, ensuring reliability and user convenience during continuous monitoring in clinical and home environments.

Overall, the study underscores the potential for integrating deep learning models into clinical practice for stroke rehabilitation. The encouraging results set the stage for future advancements in real-time rehabilitation monitoring and personalized intervention programs. Further research should focus on expanding the dataset, exploring additional data modalities, and refining model architectures to further improve classification accuracy and robustness. Specifically, a prospective study should pursue (1) prospective validation in home and community environments to establish ecological validity; (2) multi-center data collection across diverse populations, impairment levels, and devices to ensure generalizability; and (3) model adaptation strategies, such as transfer learning or lightweight personalization, to tailor models to specific patient cohorts. These steps will be essential for building robust, widely deployable systems for upper extremity movement classification in stroke rehabilitation.

## Data Availability

The raw data supporting the conclusions of this article will be made available by the authors, without undue reservation.
